# Stem cell-based ischemic stroke therapy: Novel modifications and clinical challenges

**DOI:** 10.1016/j.ajps.2023.100867

**Published:** 2023-11-10

**Authors:** Yuankai Sun, Xinchi Jiang, Jianqing Gao

**Affiliations:** a Institute of Pharmaceutics, College of Pharmaceutical Sciences, Zhejiang University, Hangzhou 310058, China; b Hangzhou Institute of Innovative Medicine, College of Pharmaceutical Sciences, Zhejiang University, Hangzhou 310058, China; c Dr. Li Dak Sum & Yip Yio Chin Center for Stem Cell and Regenerative Medicine, Zhejiang University, Hangzhou 310058, China; d Department of Pharmacy, The Second Affiliated Hospital, Zhejiang University School of Medicine, Hangzhou 310009, China

**Keywords:** Ischemic stroke, Stem cell therapy, Stem cell modification, Cell therapy challenge

## Abstract

Ischemic stroke (IS) causes severe disability and high mortality worldwide. Stem cell (SC) therapy exhibits unique therapeutic potential for IS that differs from current treatments. SC's cell homing, differentiation and paracrine abilities give hope for neuroprotection. Recent studies on SC modification have enhanced therapeutic effects for IS, including gene transfection, nanoparticle modification, biomaterial modification and pretreatment. These methods improve survival rate, homing, neural differentiation, and paracrine abilities in ischemic areas. However, many problems must be resolved before SC therapy can be clinically applied. These issues include production quality and quantity, stability during transportation and storage, as well as usage regulations. Herein, we reviewed the brief pathogenesis of IS, the “multi-mechanism” advantages of SCs for treating IS, various SC modification methods, and SC therapy challenges. We aim to uncover the potential and overcome the challenges of using SCs for treating IS and convey innovative ideas for modifying SCs.

## Graphical abstract

Schematic illustration of using SCs to treat IS. From the mechanism of SC therapy to SC modification to the challenges of SC therapy.

**Figure:**
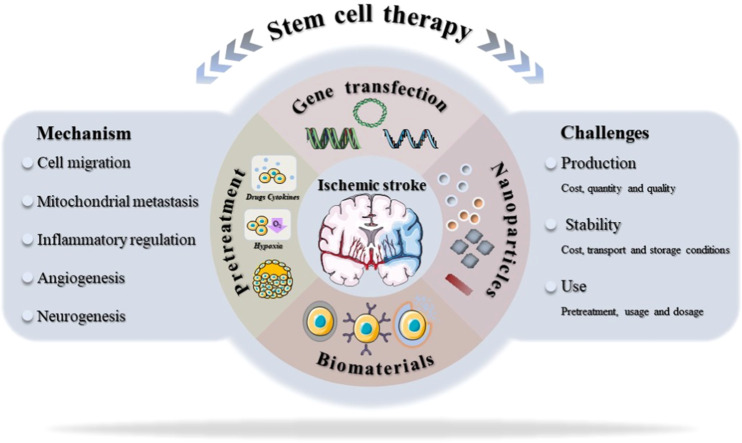

## 1 Introduction

Stroke is the second leading cause of death worldwide, begetting approximately 6.55 million deaths in 2019 [1]. The mortality rate within 30 d post-stroke can reach 24.6%, and 61.0% of stroke patients die or become disabled within 12 months, leading to severe socioeconomic burdens [2], [3], [4]. Approximately 62.4% stroke patients experienced ischemic stroke (IS) [1], which usually occurs when vascular occlusion inhibits sufficient cerebral oxygen and blood supply to the brain [4]. Due to the lengthened aging of the world population, IS incidence is increasing. Risk factors such as hypertension, hyperlipidemia, hyperhomocysteinemia, hyperglycemia, smoking and alcoholism can all give rise to IS [1,5].

Another serious problem is that IS affects more and more young adults, accounting for about 10% of total patients [6]. Following IS, intravenously administrating a tissue plasminogen activator [7] or conducting a mechanical thrombectomy [8] should be performed as soon as possible to restore blood flow to the affected area. Unfortunately, thrombolytic drugs (urokinase, streptokinase and alteplase) have weak specificity in distribution, and mechanical thrombectomy has extensive requirements regarding the patient's physical condition and hospital facility [9]. In addition, restoring the cerebral blood supply may cause a secondary reperfusion injury to the brain [10,11]. This reaction will further exacerbate reactive oxygen species (ROS) production, damage the blood-brain barrier (BBB), and cause inflammation, there by aggravating the disease. However, most neuroprotective agents only have single therapeutic effect and limited targeting ability for the brain [12]. Furthermore, the BBB prevents most drugs from entering the ischemic brain parenchyma [13]. Therefore, new agents with multi-target therapeutic effects and high targeting abilities to ischemic areas are urgently needed to meet current clinical needs.

Stem cells (SCs) can proliferate, self-renew, and differentiate into various functional cells under certain conditions [14]. Numerous studies are currently engrossed in the promising potential of SCs for treating various diseases, such as IS [15], pulmonary fibrosis [16], liver failure [17,18] and cancer [19]. Research on using multiple SC types for treating IS is extensively conducted among these studies. The strong homing, cell replacement and differentiation, and paracrine abilities of SCs have introduced superior therapeutic effects for IS. Thus far, SC therapy benefits for IS have been proven in numerous studies, and its striking therapeutic value has garnered widespread attention. Researchers have recently focused on modifying SCs to improve therapeutic efficacy and reduce toxicity [20]. These modifications usually enhance SC capabilities, such as homing properties [21], [22], [23], neural regeneration [24] and angiogenesis [25]. SC therapy provides a novel opportunity for IS treatment. There are currently 65 ongoing or discontinued clinical trials regarding SC therapy for IS, but none have been approved for clinical use (*clinicaltrials.gov*). As such, researchers must make extraordinary breakthroughs to address SC therapy limitations. Therefore, this review details IS pathogenesis, acknowledges the advantages and challenges of using SCs to treat IS, and focuses on modifying SCs to obtain improved therapeutic effects. We intend to reveal the potential and challenges of SCs in treating IS and convey innovative ideas for designing various SCs for effective IS treatment.

## 2 Overview of IS

IS is caused by the sudden blockage of the middle cerebral artery, prompting various risks, including excitotoxicity [26], inflammatory infiltration [27], oxidative stress [28] and apoptosis [29]. Due to drug and mechanical thrombolysis developments, IS treatments have considerably improved.

### 2.1 Pathological mechanisms

**Fig. 1:**
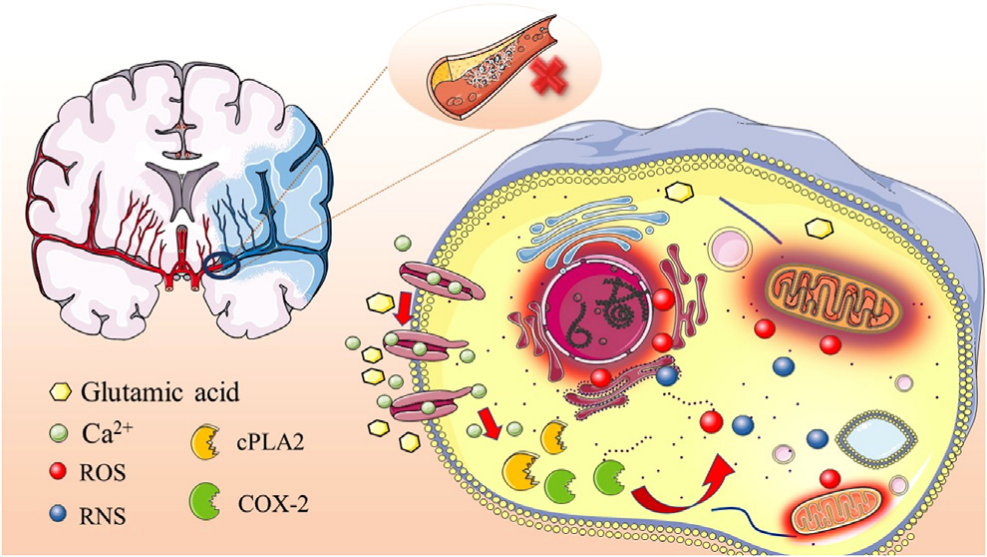
Pathological process of IS.

A series of cascade reactions emerge in ischemic areas following IS. Understanding the pathological process of IS is conducive to improving SC therapy efficacy. Due to vascular blockage, the brain experiences hypoxia and inadequate blood supply, causing oxygen and glucose deprivation in downstream tissues [30]. Two regions are typically observed: the ischemic core and the ischemic penumbra [31]. The blood supply to the core region is insufficient to maintain cell survival despite compensatory collateral circulation [32]. Cells in the ischemic penumbra can survive for a short period, and damaged brain tissue in the penumbra can recover after rapid blood flow recovery [31]. However, long-term hypoxia and glucose deprivation in the penumbra will lead to an insufficient ATP supply, encouraging abnormal ion pump and membrane depolarization [30]. From the excessive release and extracellular accumulation of glutamic acid, prolonged abnormal membrane depolarization results in pronounced excitotoxicity [33]. Specifically, over-activating glutamate-related receptors engender a large Ca^2+^ influx [34]. Subsequently, Ca^2+^-dependent enzymes greatly amplify ROS [35] and reactive nitrogen species (RNS) production [36], which in turn causes lipid peroxidation and DNA damage, stimulating the release of numerous inflammatory factors [36,37]. This process is often accompanied by mitochondrial dysfunction, inducing the release of cytochrome C and apoptosis [38] (Fig. 1).

### 2.2 Current treatment methods

Currently, the clinical treatment of IS primarily includes the following: anti-thrombotic drugs or mechanical embolectomy to recanalize blood vessels following stroke; neuroprotective agents to protect neurons from death caused by ischemia or reperfusion after revascularization (Table 1).

**Table 1:** Current treatment methods for IS.

Current treatment methods	Representative drugs	Main effects	challenges	Ref
Anti-thrombotic drugs	Clopidogrel, aspirin, heparin, warfarin, urokinase, and alteplase	Anti-platelet, anti-coagulant, and thrombolysis	Poor specificity distribution, and short treatment time window	[39], [40], [41], [42], [43]
Neuroprotective agents	Human urokininogenase, cattle encephalon glycoside and ignotin, edaravone, statins, and iron‐gallic acid coordination polymer nanodots	Anti-excitotoxicity, anti-oxidative stress, anti-inflammation	Weak penetration of blood-brain barrier	[45], [46], [47], [48], [49], [50], [51], [52]
Mechanical embolectomy	—————————	Thrombolysis	High requirements for patient status and hospital facilities	[9,44]

Anti-thrombotic drugs predominantly include anti-platelet agents, anti-coagulants and thrombolytic drugs. Because platelets are integral in creating the thrombus, anti-platelet therapy can effectively prevent thrombus formation [39]. Anti-platelet drugs, such as clopidogrel and aspirin, are generally used to treat platelet adhesion and aggregation from arterial or venous thrombosis [40], [41], [42]. Comparatively, anti-coagulants, including heparin and warfarin, are commonly administered to treat thrombotic diseases, prevent thrombosis and improve prognoses [41]. Additionally, eligible patients can be given urokinase and alteplase thrombolytic drugs under a doctor's supervision [39,43]. Unlike anti-platelet therapy and anti-coagulant therapy, thrombolytic drugs activate plasminogen, forming plasmin that directly contributes to thrombolysis. In addition, mechanical thrombectomy could enhance thrombolytic therapy. Studies have confirmed that combining mechanical thrombectomy and thrombolytic drugs achieves superior efficacy and safety than thrombolytic drugs alone [44].

Neuroprotective agents are vital in treating IS. Although they cannot restore cerebral blood flow to reduce ischemic brain injury, neuroprotective agents can counteract harmful molecular events in ischemic areas [45]. Since excitotoxicity, oxidative stress and inflammatory mediators are essential for IS progression [33], they provide a wide range of targets for neuroprotective therapy strategies. Neuroprotective agents, including human urokininogenase [46], cattle encephalon glycoside and ignotin [47], edaravone [48,49], statins [50,51] and iron‐gallic acid coordination polymer nanodots [52], exert diverse neuroprotective effects through various target mechanisms [45].

Despite the widespread use of anti-thrombotic drugs and neuroprotective agents in clinical practice, they still have certain limitations and may lead to adverse consequences. For example, thrombolytic drugs have poor specificity in distribution, hemorrhagic transformation risks, and high readmission rates for treated patients [43,53]. On the other hand, most neuroprotective agents have short half-lives, poor distribution specificity, and difficulty in crossing the BBB [39]. These challenges hinder their ability to achieve optimal therapeutic effects. Many studies are currently devoted to ascertaining safer and more effective therapies for IS to meet clinical needs.

## 3 Current status of SC therapy on IS

The clinical application of SC therapy has made remarkable progress in the past few decades, and proliferation and differentiation abilities of SCs establish the foundation for regenerative medicine [54]. Although many studies have proven the benefits of SCs in treating IS, scientists are still pursuing modifications to enhance their therapeutic effects.

### 3.1 Various SC types for therapy

Various SCs have been widely studied for IS treatment. Even though these SCs have different therapeutic mechanisms, they are pivotal in treating IS (Table 2).

**Table 2:** Current SCs used to treat IS.

Classifications	Sources	Main mechanisms	Main effects	Advantages	Challenges	Ref
MSCs	Bone marrow, adipose tissue, and peripheral blood	Paracrine effects	Anti-inflammatory, anti-apoptotic, pro-angiogenesis, and pro-neurogenesis	Multi-target therapy	Long *in vitro* amplification time, and decreased therapeutic effect after freeze-thaw	[55,^57^,[60], [61], [62], [63], [64]
NSCs	Neural tissue, and animal embryos	Neuronal differentiation, and paracrine effects	Pro-neurogenesis	Low carcinogenicity	Low survival rate, and ethical issues	[66,^67^,[69], [70], [71]
ESCs	Animal embryos, and blastocysts	Neuronal differentiation	Pro-neurogenesis	Strong differentiation ability, and strong immune regulation ability	Teratoma formation, and ethical issues	[74], [75], [76], [77], [78], [79]
DPSCs	Dental pulp nerve crest	Paracrine effects	Regulates inflammation and immune response, pro-angiogenesis, and pro-neurogenesis	Low carcinogenicity, and no ethical issues	Weak differentiation ability	[81], [82], [83], [84]
iPSCs	Human and mouse fibroblasts	Neuronal differentiation, and cell replacement	Regulates inflammation and immune response, and pro-neurogenesis	No immune rejection, and no ethical issues	Teratoma formation	[62,^73^,[86], [87], [88], [89]

#### 3.1.1 Mesenchymal stem cells

Mesenchymal stem cells (MSCs) can be derived from various tissue types, such as bone marrow, peripheral blood and adipose tissue. Currently, MSCs are the most widely studied SC group used for treating IS and other diseases. MSCs can self-renew, exhibit multi-directional differentiation, and differentiate into osteoblasts, chondrocytes and adipocytes [55]. However, considerable evidence indicates that primary beneficial effects of MSCs are not due to their ability to differentiate into tissue cells in damaged tissues [56]; instead, their value lies in their paracrine effects, which have a potent therapeutic effect on IS [56,57]. Also, MSCs can be used for autologous or allogeneic transplantation. Transplanted MSCs produce many therapeutic growth factors through the paracrine pathway, such as the vascular endothelial growth factor (VEGF) and hepatocyte growth factor (HGF) [58,59]. Interestingly, during this process, MSCs also secrete extracellular vesicles (EVs) which are rich in various therapeutic microRNA (miR-133b and miR-184) and growth factors [60,61]. These factors and EVs then exert multi-target therapeutic effects, such as anti-inflammatory, anti-apoptotic, pro-angiogenesis and neurogenesis.

The easy culture and low immunogenicity of MSCs establish them as the most commonly used SCs. However, several weeks of culture *in vitro* is required to obtained necessary number of MSCs for immune system treatment [62]. For MSCs amplified *in vitro*, cryopreservation can maintain their activity and function [63]. Unfortunately, the cryopreservation and resuscitation of MSCs may compromise their vitality, membrane integrity, and potential persistenc *in vivo* following intravenous injection [64]. Since applications of other SCs also encounter these issues, MSCs are still relatively closer to clinical application success.

#### 3.1.2 Neural stem cells

Neural stem cells (NSCs) originate from the central nervous system, and they can self-renew and differentiate into various configurations, such as neurons, astrocytes and oligodendrocytes [65,66]. Due to their ability to differentiate into neurons, NSCs play an influential neuroprotective role for IS patients. In addition, NSCs secrete various neurotrophic factors through the paracrine pathway that contributes to their therapeutic effects. For instance, NSCs can enhance the expression of brain-derived neurotrophic factor (BDNF) in a mouse model with middle cerebral artery occlusion (MCAO) [67]. Meanwhile, Yang et al. proved that the conditioned medium of NSCs improved nerve defects and reduced cerebral infarct volume while preserving the ultrastructure of mitochondria [68]. These discoveries demonstrate that NSCs can be therapeutic by secreting various nutritional factors.

After transplantation into a harsh microenvironment, only a few exogenous NSCs can survive [69]. This scenario is a common problem in SC therapy, and designing appropriate methods to improve various SC survival rates in ischemic areas is a critical area of exploration. However, the potential ethical issues of using NSCs also limit their application. Excitingly, studies have shown that NSCs have no risk of tumorigenesis [70]. NSCs obtained from mouse, rat or other animal embryonic stem cell (ESC) lines will not form tumors after transplanting into normal nude animals [71].

#### 3.1.3 Embryonic stem cells

ESCs are derived from embryos or blastocysts with potent differentiation potential [72]. ESC transplantation is an ideal method for treating neurological diseases, such as IS [73], primarily due to its differentiation into three nervous systems: neurons, astrocytes, and oligodendrocytes [74,75]. Cell therapy based on ESCs can promote nerve regeneration, reduce infarct area in mice with IS, and improve sensation and behavior recovery following stroke [76]. In addition, small EVs derived from ESCs (ESC-sEVs) have strong immunoregulatory abilities against excessive activation of the immune microenvironment by IS. ESC-sEVs can significantly reduce inflammatory cytokine, leukocyte infiltration, and neuronal death expression post-IS [77]. All these characteristics indicate that ESCs are potential therapeutic agents for IS.

However, some studies have indicated that human ESCs-derived neuronal cells have a risk of teratoma development [78]. Furthermore, ethical issues arise due to the potential destruction of embryos as the source of ESCs [79]. Although ESCs have excellent therapeutic effects, these malignant transformation risks and ethical issues limit their application; thus, their application in treating IS will be minimal.

#### 3.1.4 Dental pulp stem cells

Dental pulp stem cells (DPSCs) are pluripotent SCs around the vasculature of the dental pulp, derived from the neural crest, and can differentiate into neurons, muscles, and cartilage [80]. Some studies have reported that DPSCs can be used in treating IS, including promoting cognitive function recovery, encouraging neuron differentiation and angiogenesis, reducing infarct area, and exerting anti-inflammatory effects [81]. DPSCs’ substantial therapeutic effects on IS may be attributed to their neuronal system origin [82]. Although previous studies have verified that DPSCs can differentiate into neurons and integrate with brain tissues [83], only a few DPSCs can survive in the ischemic area, and most differentiate into astrocytes. Therefore, IS treatment is more likely to incorporate DPSCs through the paracrine pathway than cell replacement and differentiation [82]. DPSCs can also exert effective immunomodulatory properties by inhibiting over-activated T-cell responses [84].

Ethical concerns are not an issue due to the ease of isolating DPSCs from discarded teeth. Furthermore, DPSCs have no potential risk of tumor formation because of their weak differentiation ability [82,84]. Extracting DPSCs from teeth and their exosomes for IS treatment is promising for future treatment options. However, further research on treating IS with DPSCs and their early clinical transformation is needed.

#### 3.1.5 Induced pluripotent stem cells

**Fig. 2:**
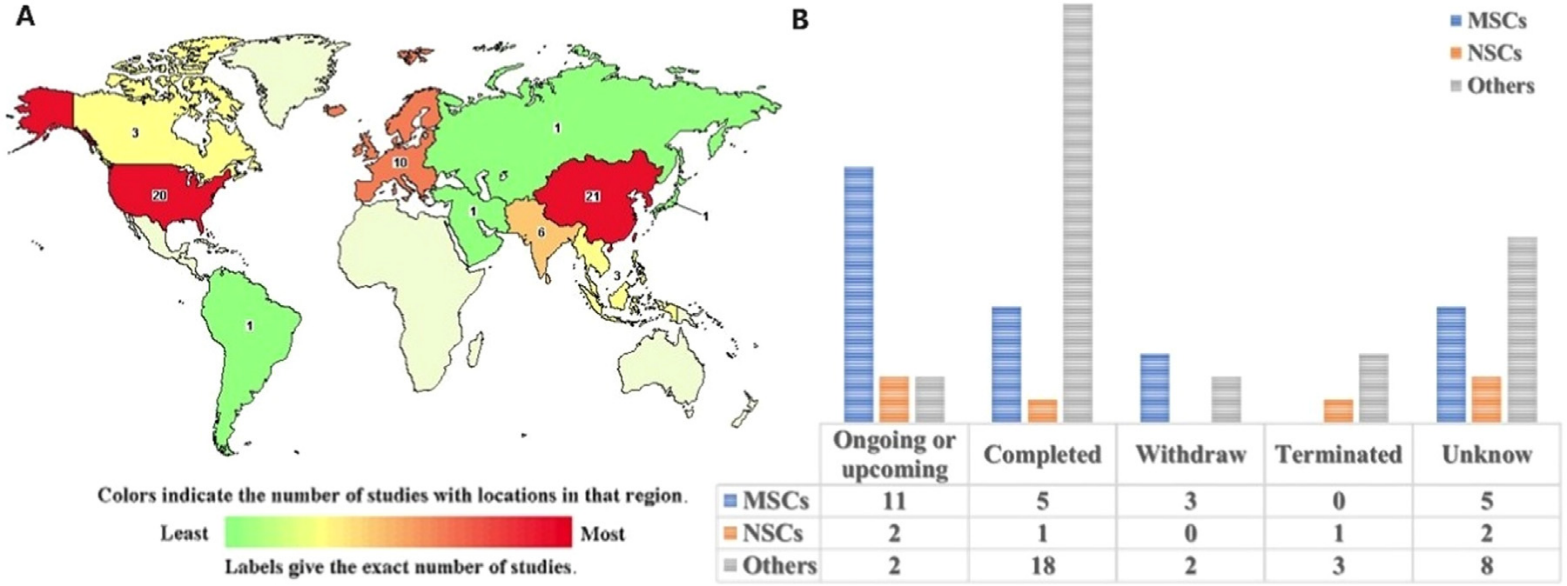
Clinical trials of SC therapy for IS. (A) Map of 65 studies found by search of stem cell | ischemic stroke. Source: https://ClinicalTrials.gov. (B) The clinical experimental status and quantity of MSCs, NSCs or other SCs.

Induced pluripotent stem cell (iPSC) technology refers to reprogramming terminal factors, first developed by Takahashi and Yamanaka in 2006 [85]. They found that by reprogramming fibroblasts from humans and mice, iPSCs similar to ESCs could be generated. The results of this study have unveiled promising pluripotent SC potential for basic research, drug research and development and SC therapy [73]. iPSCs exhibit multiple effects in treating IS, including migration to ischemic brain tissue, differentiation into neurons, pro-inflammatory factor down-regulation, and anti-inflammatory factor up-regulation, eventually improving motor function [86]. Meanwhile, iPSCs enhance the thrombolytic effect of low-dose tissue plasminogen activators [87]. A recent study found that small EVs derived from iPSCs can rejuvenate the blood-brain barrier of aged mice, preventing IS occurrence [88]. Interestingly, NSCs derived from iPSCs (iNSCs) can differentiate into neurons, oligodendrocytes and astrocytes, exhibiting considerable neuroprotective effects [89]. In addition, other SCs derived from iPSCs displayed the same therapeutic effect in the MCAO rat model, including regulating inflammation and immune response and promoting nerve differentiation [73]. iPSCs originate directly from patients and behave similarly to ESCs, overcoming immune rejection and ethical issues [62,73]. Unfortunately, studies have reported that the MCAO rats formed teratomas four weeks after iPSC transplantation [90], the primary reason limiting its clinical application. In recent years, using pluripotent cell-specific inhibitors before transplantation can significantly inhibit teratoma formation, improving iPSC safety in future IS treatments. Currently, there are 65 clinical trials for SC treatment of IS, with more than half of the clinical trials concentrated in China and the United States (Fig. 2A). Among them, there are 15 ongoing or upcoming clinical trials, including eleven for MSCs and two for NSCs, which further illustrating the scientific interest and advantages of implementing MSCs and NSCs for IS treatment (Fig. 2B). However, most clinical trials concerning other SCs have been completed or withdrawn, and further investigation is needed to determine their effects. (Source: https://ClinicalTrials.gov)

### 3.2 Therapeutic mechanisms of SCs in IS

SCs’ neuroprotective effects on IS have been investigated in many studies and predominantly involve cell migration, cell differentiation, cell substitution, paracrine effects, and other aspects, such as promoting mitochondrial metastasis.

#### 3.2.1 Migration

SCs are widely studied in treating IS because they naturally target ischemic-damaged areas [91]. SCs must first cross the BBB to reach these ischemic regions. The BBB consists of a basal layer, peripheral cells and astrocytes, that are capable of maintaining the brain's steady microenvironment. An accurate mechanism for SCs crossing the endothelial cell layer and being recruited into the ischemic tissue has yet to be established. Still, increasing evidence suggests that the interaction between stromal cell-derived factor-1α (SDF-1α) and its receptor chemokine receptor C-X-C chemokine receptor 4 (CXCR4), which is highly expressed on the surface of SCs, is essential for controlling cell migration [92,93]. After a stroke, large quantities of SDF-1α are released in the ischemic area, which has a robust recruitment effect on SCs expressing CXCR4, and achieves the target outcome. Interestingly, studies have found that the hypoxia-inducible factor-1 (HIF-1) can regulate SDF-1 gene expression [94]. Augmenting HIF-1α expression can improve survival rates of SC in ischemic regions [95], which partly explains why certain hypoxic preconditioning can enhance SC homing.

Currently, many targeted therapies for IS are based on the SDF-1α-CXCR4 axis. Unfortunately, neutrophils and macrophages are recruited to the ischemic area alongside SCs through the SDF-1α-CXCR4 axis, aggravating inflammatory infiltration [96,97]. Therefore, Shi et al. co-incubated SCs with Fe_3_O_4_ to enhance the expression of CXCR4 on SC membranes, which was used to construct the biomimetic carrier. The biomimetic carrier with CXCR4 overexpression concurrently achieved the targeted therapy and cut off inflammatory cell recruitment in ischemic areas [98]. Other signals influence SC homing, such as the c-MET signal [99]. In addition, brain inflammation reduces the protection for the BBB's tight junction integrity, allowing SCs to cross the BBB through paracellular pathways by shape changes [100].

In conclusion, active homing to the ischemic area is the premise and critical factor for SCs to exert their therapeutic effects. Furthermore, enhancing the homing abilities of SC will expand their therapeutic capabilities, which is a prominent topic in SC modification.

#### 3.2.2 Differentiation and substitution

The neural repair function of SC is directly reflected in their differentiation into new nerve cells and the replacement of damaged nerve tissue, which ensures the integrity of nerve conduction pathway [62]. Due to their strong differentiation ability to restore normal nerve conduction, NSCs can replace damaged neurons, astrocytes or oligodendrocytes. Exogenous SC transplantation can also achieve this neuroprotective effect by compensating for the loss of nerve cells induced by differentiation. Additionally, previous studies have proven that SCs’ homing to ischemic regions will differentiate into mature neurons, forming a novel neural circuit [62,66]. This potent neuronal differentiation is demonstrated by over 50% of SCs expressing neuronal phenotypes two months post-transplantation [101].

While differentiating into neurons, SCs located in ischemic areas can also repair the damaged BBB by interacting with peripheral cells, endothelial cells and astrocytes to accelerate neural circuit reconstruction [102]. Although the exact neural differentiation mechanism of SCs has not yet been universally recognized, the Wnt/β-Catenin signaling may be a prominent factor in SC self-differentiation [103]. This pathway can promote the differentiation of NSCs into neurons rather than astrocytes [104].

In summary, neurogenesis caused by cell differentiation and neural circuit reconstruction from cell replacement may be the mechanism by which SCs improve neurological function following IS. One of the main reasons SCs cannot be used to treat IS is their insufficient neural differentiation ability.

#### 3.2.3 Paracrine secretion

SCs can accelerate the recovery of neural function by secreting various therapeutic factors, including EVs, growth factors, chemokines and cytokines [105]. SC paracrine functions are reflected in the followings:

1. SCs can promote neurogenesis by secreting neurotrophic factors, such as nerve growth factor (NGF), insulin-like growth factor 1 (IGF-1), glial cell-derived neurotrophic factor (GDNF) and BDNF [106,107]. For example, BDNF can advance neurogenesis by interacting with the tyrosine kinase receptor [108].

2. SCs can encourage angiogenesis by secreting pro-angiogenic factors, such as Notch 1, basic fibroblast growth factor (bFGF), HGF, angiopoietin-1 (Ang-1), Ang-2 and VEGF [62,^105^,109]. For example, VEGF can promote angiogenesis by fostering the formation of immature blood vessel and the migration and proliferation of endothelial cell [109].

3. SCs can exert influential immunomodulatory and inflammatory regulatory effects by improving inflammatory factor secretion [110]. Studies have indicated that SCs can secrete the crucial transforming growth factor-β (TGF-β) which alleviates the immune response in ischemic brain tissue [111], reducing the elevated levels of monocyte chemoattractant protein-1 (MCP-1) caused by cell death in the infarcted region and inhibiting the massive infiltration of CD68^+^ immune cells from BBB damage. In addition, SCs can regulate the inflammatory environment by up-regulating anti-inflammatory cytokines and down-regulating pro-inflammatory cytokines. For example, SCs regulate the inflammatory environment by increasing IL-10 expression and decreasing tumor necrosis factor-α (TNF-*α*), interleukin-1β (IL-1β) and IL-6 expression [105,112]. Interestingly, the proliferation of SC in the ischemic area is stimulated by regulatory T cells (Tregs), but blocking IL-10, an anti-inflammatory cytokine, will affect this process [113].

4. SCs inhibit neuron apoptosis in ischemic regions by up-regulating anti-apoptosis (*i.e.*, Livin) and down-regulating pro-apoptosis (*i.e.*, Caspase-3) protein secretion [62]. Meanwhile, studies have demonstrated that SCs can also inhibit apoptosis by secreting granulocyte-macrophage colony-stimulating factor (GM-CSF), B lymphocyte-2 (Bcl-2) and other molecules [114,115]. Among them, the hematopoietic growth factor GM-CSF can improve cell survival. In addition to the direct anti-apoptosis effect, SCs secrete several neurotrophic factors, such as BDNF, VEGF, and the previously mentioned neurotrophic factors, to enhance neuron survival.

SCs’ paracrine functions further establish them as favorable “drugs” for treating IS. Thus, researchers consistently pursue SCs to improve their paracrine ability and maximize their therapeutic advantages.

#### 3.2.4 Mitochondrial metastasis

In addition to the above mechanisms, SCs can promote neural function recovery through mitochondrial metastasis. After a stroke, numerous mitochondria in nerve cells will be damaged, triggering mitochondrial autophagy to ensure mitochondria quality [116]. Removing damaged mitochondria through mitochondrial phagocytosis will reduce ROS generation and improve the harsh microenvironment in ischemic areas [117]. ROS refers to oxygen-containing and active substances *in vivo*, and excessive ROS induces lipid peroxidation and cell damage.

Although this partly improves the microenvironment, excessive mitochondrial autophagy may prompt the digestion and death of damaged neuron [116]. Based on these observations, transferring healthy mitochondria to damaged cells is a promising potential treatment [118]. Many studies have substantiated that SCs can act as mitochondria donors to maintain cell mitochondrial equilibrium [119]. Mitochondrial metastasis of SCs depends on forming Cx43-regulated gap junction channels [119,120]. The mitochondrial transfer efficiency of SCs with enhanced Cx43 expression is almost twice that of normal SCs, while that of SCs with silenced Cx43 gene expression hardly occurs [16].

In conclusion, delivering healthy mitochondria to damaged cells in ischemic areas is a promising treatment technique, and SCs have excellent mitochondrial delivery capabilities. However, research on how to expand mitochondrial delivery efficacy with SCs is still in the early stages. Thus, identifying multiple signaling pathways for the mitochondrial delivery of SCs is essential.

### 3.3 Modification methods

#### 3.3.1 Modification by gene transfection

Gene transfection has been extensively incorporated into genome function and gene therapy research [121]. This technique modulates gene expression by transferring specific genes into cells, ultimately enhancing or inhibiting specific functions [122]. Gene transfection for SCs is primarily utilized to improve their therapeutic ability. For example, VEGF gene transfection can enhance SCs’ vascular regeneration ability [123]. Some miRNAs, such as miR-124, also improve SC therapeutic function by promoting their differentiation into mature neurons [124]. Furthermore, CXCR4 gene transfection can be performed on SCs to enhance their homing ability [23].

**Fig. 3:**
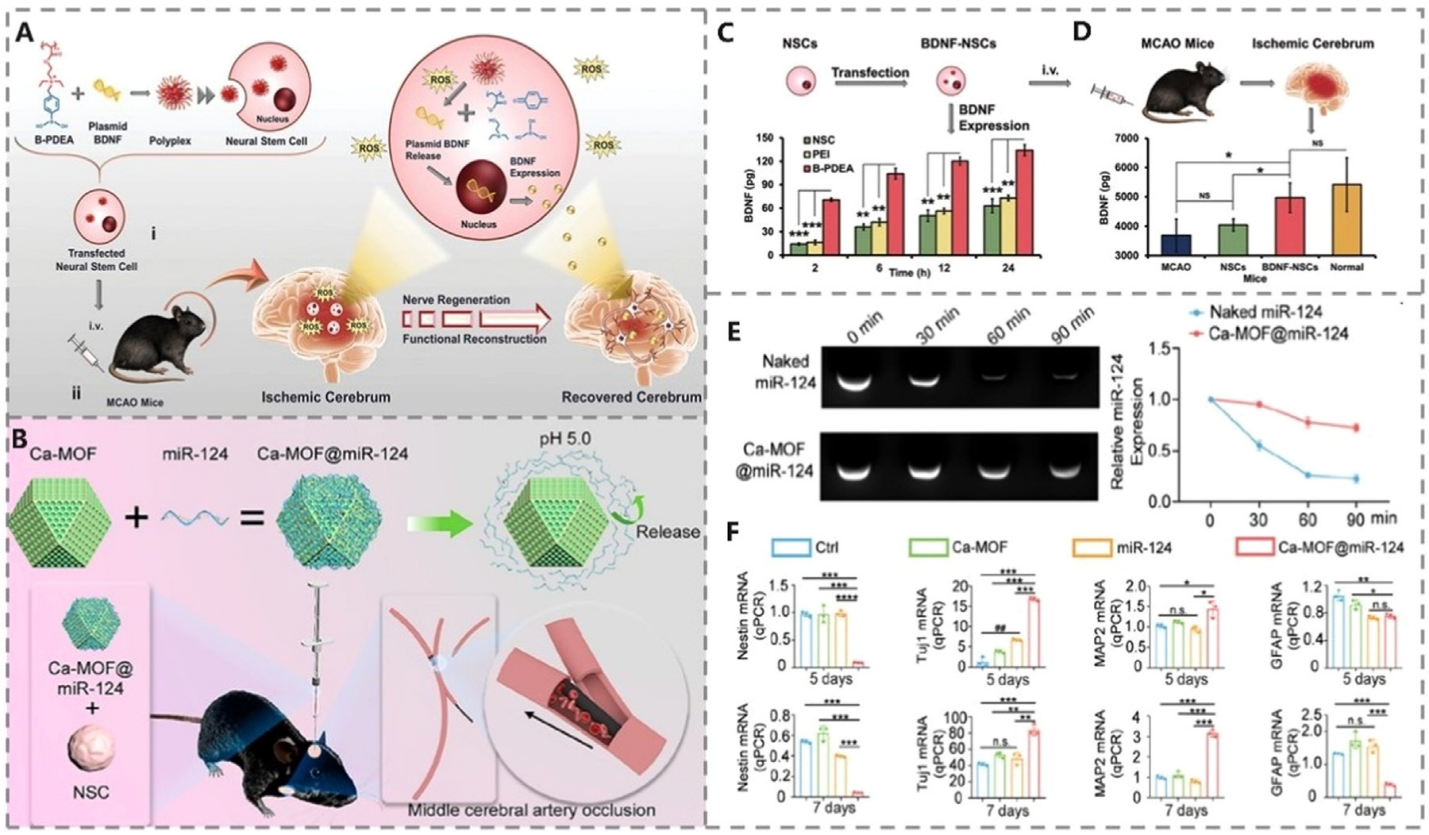
Gene transfection of SCs. (A) Schematic diagram of ROS responsive B-PDEA as gene vector for BDNF transfection. (B) Schematic diagram of nuclease protective Ca-MOF as gene vector for miRNA-124 transfection. (C) Cumulative expression of BDNF in NSCs transfected with B-PDEA or PEI. (D) The total amount of BDNF in brain homogenate of MCAO mice after different stem cell therapy. Reprinted with permission from [24]. Copyright 2019 Wiley-VCH. (E) The expression of miR-124 in Ca-MOF@miR-124 or naked miR-124 during simulated nuclease degradation. (F) The expression of neuronal differentiation markers and glial differentiation markers of NSCs in 5 or 10 d under different treatments. Reprinted with permission from [125]. Copyright 2022 American Chemical Society. The data were presented as the mean ± SD.

Using bio-responsive materials as gene carriers to enhance SC therapeutic effects has also been widely exercised. For instance, we used ROS-responsive material poly [(2-acryloyl) ethyl (p-boronic acid Benzyl) diethyl ammonium bromide] (B-PDEA) as a BDNF gene carrier to transfect NSCs [24] (Fig. 3A). BDNF then interacted with tyrosine kinase receptors to promote neuron survival [108]. The results demonstrated that B-PDEA transfection increased BDNF secretion from SCs (Fig. 3C) and its amount in brain homogenate (Fig. 3D). Yang et al. produced a non-viral gene vector, calcium-metal organic framework (Ca-MOF), and utilized it for SC miRNA delivery to obtain a better therapeutic effect on IS [125] (Fig. 3B). Ca-MOF protects the target gene (Fig. 3E) and aids miRNAs in guiding SC neural differentiation (Fig. 3F).

Conditions should be carefully considered during SC gene transfection. Studies have highlighted that cell morphology influences transfection efficiency [126]. The expansion and elongation of cell morphology benefit transfection, and well-extended SCs have a higher rate of transfection efficiency. The larger the adhesion area, the higher the transfection efficiency. In contrast, the effect of cell diffusion area on transfection efficiency is relatively small [127].

Moreover, the choice of vector significantly affects SC gene transfection. Virus-mediated transfection is a widely used gene modification method in clinical practice [121,128]. Although high transfection efficiency and convenient use are advantages of virus vector [121], the immunogenicity and cytotoxicity limit their application. Non-viral vectors have been extensively studied to avoid security problems, including mesoporous silica and gold nanoparticles based on silica systems [129,130]. These materials lack mutagenicity but induce pro-inflammatory reactions and low transfection efficiency. These factors result in a trade-off when selecting transfection vectors, and scientists are expected to make further breakthroughs to achieve high transfection efficiency while avoiding cytotoxicity.

#### 3.3.2 Modification by inorganic nanoparticles

**Fig. 4:**
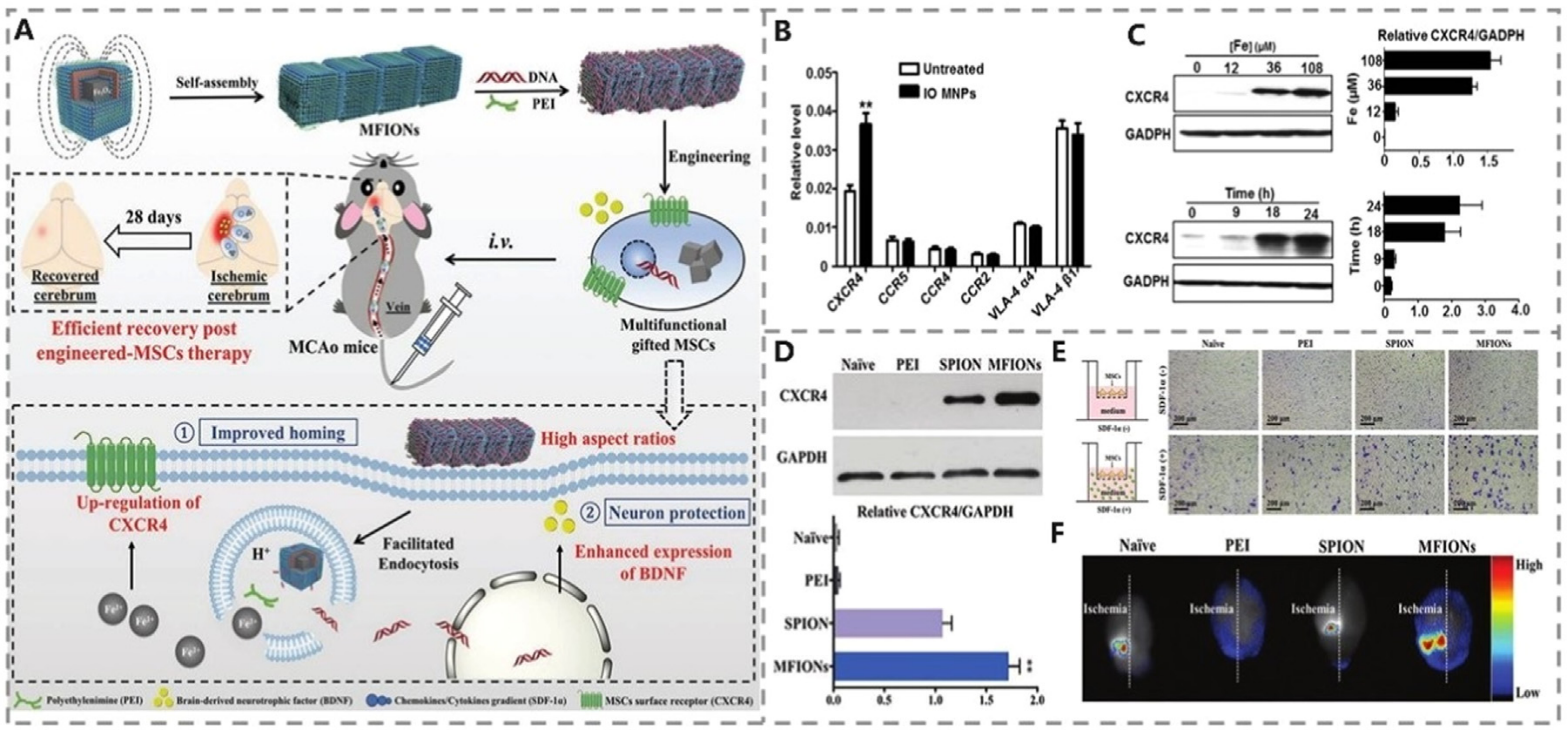
Modification by nanoparticles of SCs. (A) Schematic diagram of MSCs engineering based on MFION for IS treatment. (B) The expression of MSCs surface receptors after 2 h incubation with or without IO MNPs. (C) The expression of MSC CXCR4 after incubation with different concentrations of IO MNPs for different time. Reprinted with permission from [134]. Copyright 2014 American Chemical Society. (D) The expression of MSC CXCR4 after treated with different nanoparticles. (E) The migration ability of MSCs treated with different nanoparticles to SDF-1α. (F) The homing ability of MSCs treated with different nanoparticles to the ischemic brain. Reprinted with permission from [21]. Copyright 2019 Wiley-VCH.

Numerous nanoparticle-based drug/gene delivery systems have been developed [131,132]. Using nanoparticles to modify SCs to obtain a better therapeutic effect for IS has been confirmed by many studies. Research on nanoparticle-modified SCs mainly focuses on improving SCs’ homing ability. After a stroke, CXCR4 overexpression on SCs will improve cell homing efficiency *via* the SDF-1α-CXCR4 axis [99,133]. Various iron-based magnetic nanoparticles (MNPs) have positively enhanced CXCR4 expression [134] (Fig. 4B and 4C); however, external magnetism optimization is usually required to increase SC absorption of these iron-based MNPs [135].

Based on this limitation, we invented a magnetosome-like 1D ferromagnetic iron oxide nano-chain (MFION) (Fig. 4A), a chain-like non-viral vector beneficial for cell uptake without external magnetic force [21]. At the same time, MFION overexpresses CXCR4 on SCs, enhancing SC homing in ischemic areas (Fig. 4D-4F) and therapeutic effects. Moreover, SCs treated with MNPs can target ischemic areas through external magnetic attraction [136]. These iron-based nanoparticles can also be used as gene vectors for gene transfection, including superparamagnetic iron oxide nanoparticles (SPION) and MFION.

Nanoparticles have also been modified to enhance other SC capabilities. For instance, Tang et al. developed a melanin nanoparticle that enhances MSCs’ therapeutic ability against hypoxic-ischemic injury by up-regulating antioxidant defense and inhibiting apoptosis [137]. Interestingly, nanoparticle-modified SCs can enhance survival [95,138] and neural differentiation abilities [139] during IS therapy. Among them, nanoparticles formed through functional peptide and SPION self-assembly were delivered into cells, and functional peptides up-regulated HIF-1α and induced a higher NSC survival rate. In addition, Fe_3_O_4_ nanoparticles are used as a “bridge” to connect the antioxidant layer with SCs, achieving a higher SC survival rate.

Understanding transplanted SC migration, distribution, and survival *in vivo* is significant for basic research and clinical SC transformation. Nanoparticles are also widely used for long-term SC tracking in treating IS. For example, magnetic resonance imaging (MRI) can accurately monitor the *in vivo* behavior of MSCs labeled with iron-based MNPs [134]. Recently, iron oxide nanoring has been reported as a tracer [140], effectively labeling MSCs by locally inducing heat-enhanced membrane permeability for MRI tracking and targeted IS therapy. Furthermore, Xu et al*.* designed a dynamic and enhanced dual-mode tracking system to improve the high ROS microenvironment and monitor the *in vivo* fate of MSCs during long-term IS treatment [138]. Unlike other nanoparticle modifications, the nanoparticles in this study were anchored to the SC membrane but not internalized into the SCs. In addition, the dynamic fate of SCs can be monitored for up to 28 d Magnetic nanobubbles (MNBs) can be used for long-term SC tracking. Li et al*.* assembled MNPs into MNBs, which were then internalized by NSCs to realize MRI and ultrasonic imaging monitoring [139]. Meanwhile, MNPs can direct NSCs to differentiate into neuronal phenotypes by up-regulating the BMP2/Smad signaling pathway.

In short, various nanoparticles enhance the therapeutic abilities of SCs and enable tracking of their behavior *in vivo*. Further development of functional nanoparticle is anticipated to enhance the therapeutic benefits of SC in IS treatment.

#### 3.3.3 Modification by biomaterials

Several studies have verified that biomaterials can regulate SC behavior, such as polypeptides [141] and hydrogels [142]. Modifying the surface of living cells with natural or synthetic special functional materials has yielded novel research prospects in the biomedical field [143]. Currently, biomaterial modification aims to strengthen SC homing ability or survival rate in the ischemic area.

**Fig. 5:**
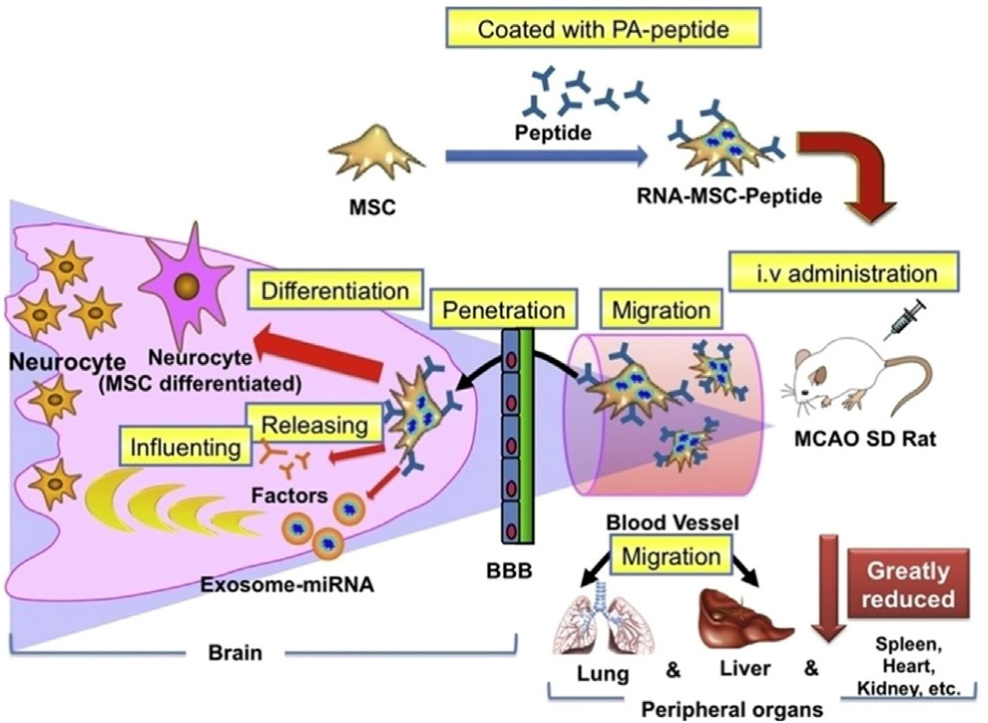
Modification by biomaterials of SCs. Reprinted with permission from [141]. Copyright 2017 Elsevier.

Certain biomaterials can enhance the homing ability of SCs. For instance, our study demonstrated that coating palmitic acid-peptides on MSCs can improve guidance, increase MSC numbers in ischemic tissues, and reduce distribution in surrounding tissues [141] (Fig. 5). In another study, lipid-PEG (lipo-PEG)-linked recombinant CXCR4 non-invasively covered on the surface of MSCs to treat ischemic cardiomyopathy [143]. Although this surface modification has not been applied for IS treatment, it improved MSCs’ gradient migration to SDF-1, suggesting its potential to enhance the therapeutic ability against IS. Overall, modifying SCs with biomaterials has been proven to improve the targeting ability of SCs to ischemic regions.

Meanwhile, biomaterial modification of SCs can improve their survival rate in ischemic areas. Recently, Xu et al*.* addressed the low NSC survival rate in ischemic regions by designing a lipid microcapsule to induce autophagy [144]. The lipid microcapsules provided physical barriers and enhanced autophagy flux for NSCs, reducing infarct volume, lessening brain edema, and ultimately improving the survival rate of model mice. Many recent studies have focused on anti-ROS materials to protect SCs from damage. For example, an injectable PEG hydrogel that can degrade ROS has been designed to enhance SC retention and antioxidant protection [142].

Various biomaterials have been developed to enhance the homing ability of SCs and their survival rate in ischemic areas. The development of more functional biomaterials to enhance the therapeutic ability of SCs is anticipated.

#### 3.3.4 Modification by pretreatment

**Fig. 6:**
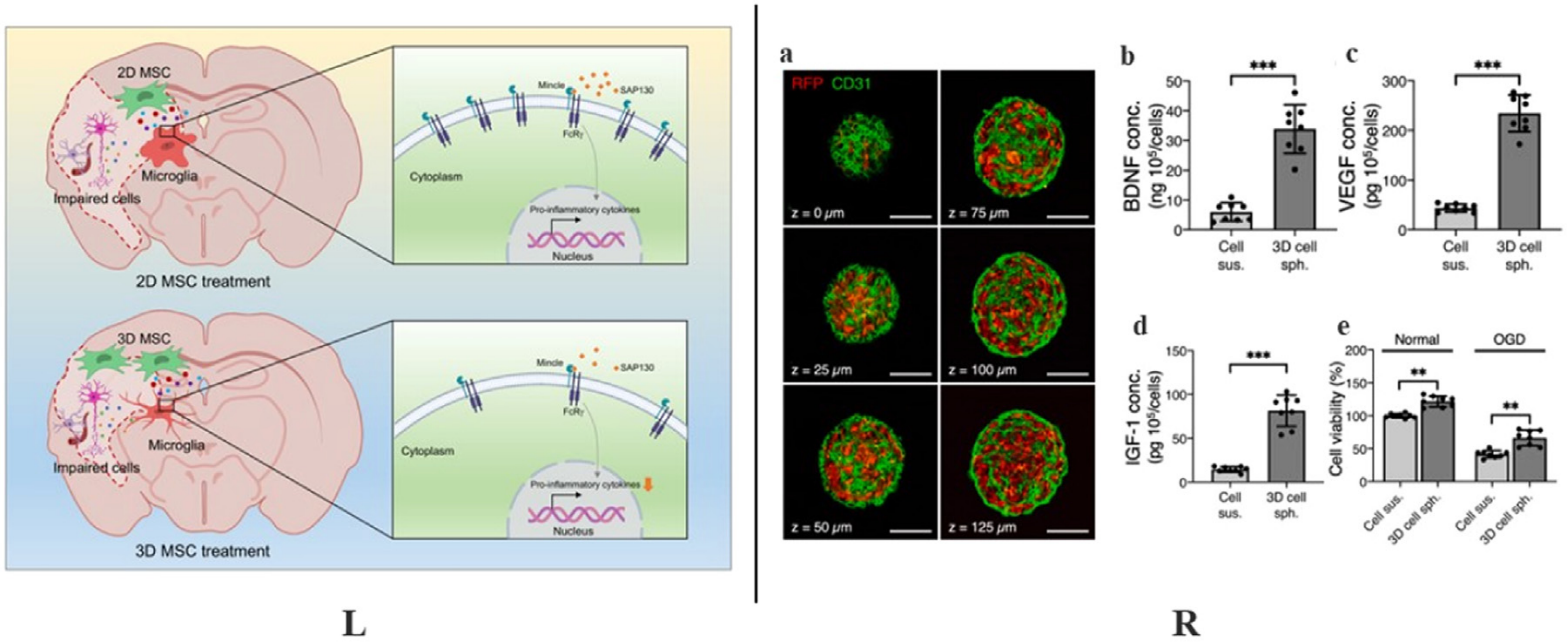
Pretreatment of SCs. The potential role of SCs in 3D culture or 3D spheroids. (L) The potential mechanism of 3D-MSC exerts stronger therapeutic ability. Reprinted with permission from [145]. Copyright 2021 Springer Nature. (R) (a) Representative confocal Z-stack images of a 3D cell spheroid mixing MSCs (Red) and HUVECs (Green). The concentration of (b) BDNF, (c) VEGF, and (d) IGF-1 in cell suspension (cell sus.) or 3D cell spheroids (3D cell sph). (e) Cell viability in cell sus. and 3D cell sph. cultured under normal conditions or oxygen and glucose deprivation conditions. Scale bars, 100 μm. Reprinted with permission from [150]. Copyright 2021, Elsevier.

Different treatment conditions and culture environments can alter SC characteristics and therapeutics *in vitro* and *in vivo*. Recently, there have been many studies on the three-dimensional (3D) culture [145] and various pretreatment of SCs [146]. Compared with traditional culture methods, 3D culture significantly improves SCs’ therapeutic effect on IS [145]. The most significant difference from 2D culture is that 3D uses a customized 3D artificial matrix to simulate a natural environment for SCs by better reflecting the intracellular environment [147]. The most direct manifestation is that cell adsorption will occur over the entire cell surface in 3D culture, while cell adsorption only occurs on the side of cells in contact with the 2D culture surface. Studies have shown that compared to SCs cultured in 2D, 3D-cultured SCs through tail vein injection significantly reduce pro-inflammatory cytokine levels, microglia and cerebral infarction volume, simultaneously increasing SCs in ischemic regions [145]. At the same time, RNA sequencing of microglia at the lesion site proved that 3D-cultured MSCs exhibited a more substantial therapeutic effect on IS, potentially by inhibiting microglia activation (Fig. 6L). In addition, assembling SCs into 3D multicellular spheroids can improve their paracrine effects, benefiting transplanted cells' survival and therapeutic effects [148], [149], [150]. Hsu et al*.* demonstrated that assembling MSCs and human umbilical vein endothelial cells (HUVECs) into 3D spheroids strikingly improved cell viability and retention [150]. The transplanted 3D spheroids had significant neuroprotective, angiogenesis-promoting and anti-scarring abilities compared to MSCs/HUVECs suspensions. This 3D spheroid promotes levels of paracrine factor expression and secretion, eventually yielding remarkable brain structure and motor function recovery in MCAO mice (Fig. 6R).

SC pretreatment, often involving hypoxia, before transplantation has been widely studied in recent years to improve the curative effect [146]. For example, the most direct influence on hypoxic preconditioning MSCs (HP-MSCs) is increased migration-related protein expression (such as CXCR4) [151]. Compared to MSCs cultured in normal oxygen, HP-MSCs displayed enhanced migration ability to ischemic areas and performed better in the adhesion removal test during sensory-motor function determination. Hu et al. listed the effects of a hypoxic culture environment with different oxygen content in SCs [146], including 1% oxygen content to prevent MSC apoptosis [152] and 2% oxygen content to reduce MSCs’ tumorigenic potential [153].

**Fig. 7:**
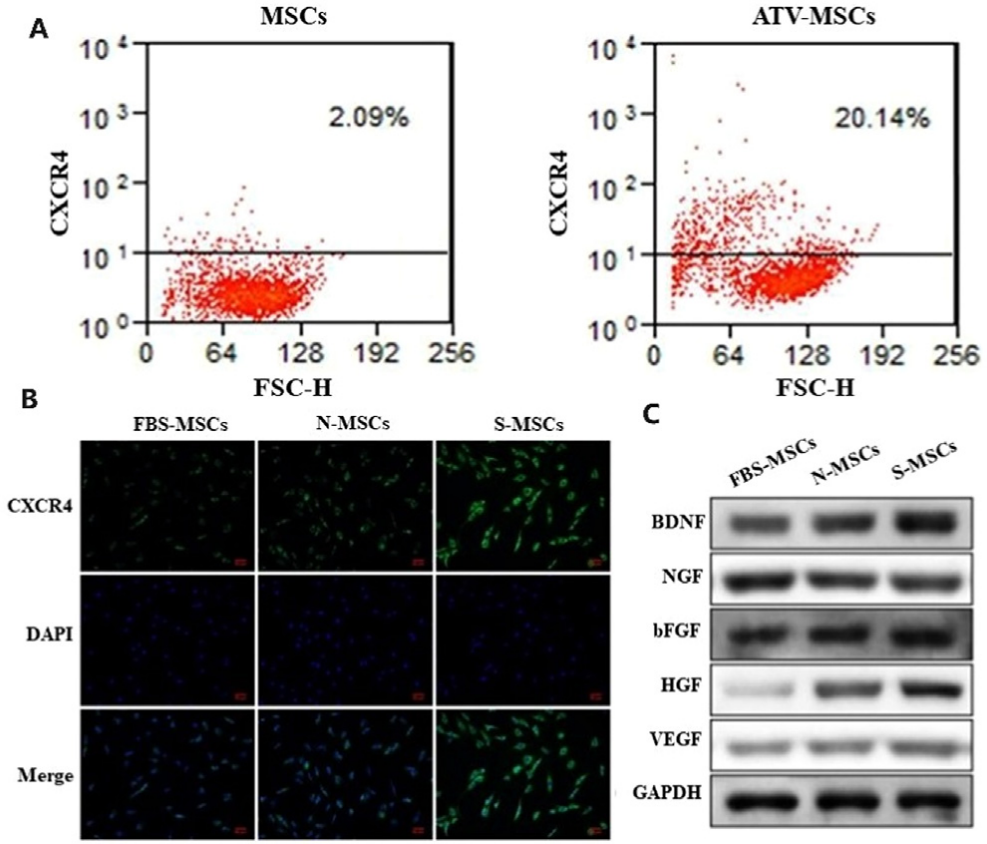
Pretreatment of SCs. (A) The flow cytometry results of MSC CXCR4 pretreated with or without atorvastatin. Reprinted with permission from [22]. Copyright 2022 Springer Nature. (B) The expression of CXCR4 on the surfaces of MSCs without pretreatment (FBS-MSCs), MSCs pretreated with normal brain tissue (N-MSCs), and MSCs pretreated with ischemic brain tissue (S-MSCs). Scale bar: 50 µm. (C) The expression of various paracrine factors of FBS-MSCs, N-MSCs and S-MSCs. Reprinted with permission from [156]. Copyright 2022 MDPI.

Meanwhile, studies have shown that 0.1%−0.3% oxygen will promote growth factor secretion, including VEGF, GDNF and BDNF, promoting neurogenesis and nerve function recovery [154]. Our recent work [155] found that hypoxic preconditioning of NSCs can regulate miRNA expression levels encapsulated in exosomes to enhance IS therapeutic benefits, further illustrating the broad therapeutic advantages of hypoxic-pretreated SCs. In addition to hypoxic preconditioning, ischemic brain tissue preconditioning regulates *in vivo* SC behavior, including promoting CXCR4 expression to facilitate homing and growth factor release [156] (Fig. 7B and 7C).

Pretreating SCs with certain drugs will also improve their curative effect. For example, pretreating MSCs with atorvastatin can enhance their homing ability by regulating miR-124a/CXCR4 signaling [22] (Fig. 7A). Similarly, some cytokine pretreatments, including IL-1β, can improve MSC migration by increasing various cytokine (TNF-α), chemokine (CXCL1), and adhesion molecule (intercellular cell adhesion molecule-1 (ICAM1)) expression in MSCs [157]. Although some drugs or cytokine preconditioning have not yet been applied, these preconditioning schemes may improve IS treatment. In summary, SC pretreatment (such as ischemia, hypoxia, drugs, and cytokines, may be an effective strategy for treating cerebral ischemic injury.

## 4 Challenges and prospects

With the in-depth research of SC therapy for IS, the importance of improving production efficiency, quality, and safety of SC products must also be emphasized.

### 4.1 Challenges in production

Due to the rapid cell therapy development, there is an unprecedented demand for SCs, and the annual MSC usage is nearly 300 trillion [158]. The provided data indicates that successful SC therapy requires approximately 1 × 10^9^ cells, but the original amount of The collection of SCs is limited. Collecting SCs is a mature industrial project; therefore, a significant number of SCs must be obtained through adequate *in vitro* expansion to meet the cell demand required for clinical application. However, this *in vitro* expansion is time-consuming and takes several weeks [62]. Therefore, it is crucial to develop novel culture technologies for faster *in vitro* expansion of SCs for effective clinical therapy applications [159].

Quality must be considered when creating SCs. Good Manufacturing Practice (GMP) ensures quality control in drug production and minimizes risks. Some scholars have detailed the production of GMP-grade SCs for treatment [159], [160], [161]. This process primarily includes cell identification, viability, growth activity, purity, uniformity, abnormal immunological reactions, tumorigenicity, biological efficacy tests, sterility tests, and detecting mycoplasma, intracellular and extracellular pathogenic factors, endotoxins, residual amounts of culture medium and other additives. GMP-grade SC production is time-consuming and expensive; however, it significantly improves the quality and safety of SC treatment. This requirement necessitates considering how to reduce production costs and ensure quality in relation to rapid SC production.

Many SC enterprises have emerged recently, including Beike Bio and SinoCell Technology Ltd. These companies manage cell collection, processing, storage, and distribution, forming a complete SC industry chain. In addition, various large SC enterprises have developed a large-scale SC preparation process based on 3D microcarriers to meet the demand for high-quality and large-scale SC production. SCs are cultured in a mimetic physiological microenvironment formed by the 3D microcarriers, which ensures essential quality attributes of cells and preparation stability. However, how to completely remove 3D microcarriers during large-scale preparation while adhering to safety standards for clinical application remains an issue.

### 4.2 Challenges in stability

SC storage and transportation conditions will significantly affect their stability. Produced SCs will be transported in a cold chain at 4–10 ℃ to maintain their vitality, which increasing costs. Most hospitals are likely to prepare SCs on-site during clinical trials [160]. However, if the demand for SCs as a standard treatment method increases, hospitals may not be able to maintain their self-sufficient production of SCs, and thus would still need external manufacturers for the supply of specific, making transportation of SC inevitable. Although cryopreservation at −70 ℃ to −196 ℃ can maintain the activity and functional stability of SC [63], transporting them at extremely low temperatures using liquid nitrogen is expensive, and the cryopreservation reagent DMSO is potentially toxic.

Transportation under ambient conditions may be a viable solution. A previous study indicated that mammalian cells directly suspended in a culture medium maintained high viability after being transported from Britain to China over 36 h at ambient temperature. These results were considerably better than transportation with ice packs [162]. Interestingly, forming SC spheroids can protect SC activity and facilitate long-term storage and transportation at ambient temperature [163]. However, SC spheroid formation requires substantial time, financial resources, and increasing processing costs before use. Therefore, further optimization is necessary for the storage and transportation conditions of SCs in order to establish lower transportation cost and ensure the stability of SC.

### 4.3 Challenges for use

#### 4.3.1 Pretreatment before use

Even if the aforementioned restrictions are overcome, specific professional operation is still required for SC therapy. For example, SCs stored at low temperatures must be reheated before use to restore activity. Unfortunately, cryopreservation and resuscitation before use will destroy the vitality and cell membrane of SCs, potentially reducing persistence *in vivo* following intravenous injection [64]. Also, special attention should be paid to the reheating rate during this operation in order to ensure the survival of frozen SC. Generally speaking, the faster the reheating speed, the better it is. Ultimately, improving the survival rate and activity of SCs during processing remains a problem necessitates discussion.

#### 4.3.2 Route of administration

There is no unified view on the exact SC administration route, although most approaches for IS treatment research are intravenous injections. Compared with other administration routes, intravenous injection is the simplest and safest method, exhibiting the lowest invasiveness [160]. However, only a few cells can reach schemic areas of the brain through this method, and cell accumulation in non-target organs becomes an issue. Studies have proven that intracranial injection can directly deliver SCs to ischemic areas and has an excellent therapeutic effect on IS [164]. Still, its considerable invasiveness and risks of causing additional brain damage limit its use. In addition, some studies have proven that arterial injection is more effective than intravenous infection in transporting cells to the ischemic hemisphere [165]. However, additional ischemic injuries will occur if the cell cluster blocks the artery. It is essential to note that the adverse effects of the injection operation should not be underestimated, as SCs may die immediately due to excessive force during the injection [166]. Optimizing the administration route or strengthening the ``shield” of SCs should be a focus in studies.

#### 4.3.3 Dosage

The viewpoint that “the more SC transplants, the better” is incorrect. Studies have proven that transplanting additional SCs does not improve cell survival or have substantial therapeutic effects in the microenvironment [167]. In addition, excessive administration of single-dose may cause vascular embolism risks [168]. Although the transplantation quantity should not be as high as possible, it is necessary to consider that specific SC concentrations are required to produce the targeted therapeutic effect. Notably, different MSC doses may cause different therapeutic effects on IS. Compared with other doses, a low dose (1 × 10^6^ cells) is more able to restore neurological function, and a high dose (2 × 10^7^ cells) has a stronger ability to inhibit microglia activation [169]. Furthermore, due to the individual differences of patients and disease courses, giving all patients the same amount of SCs is impossible. This observation reminds us that transplanted SC concentrations should be neither too low nor too high, and it is necessary to optimize the individualized treatment dose and establish a more accurate dose relationship.

#### 4.3.4 Treatment time window

Controlling time is also pivotal to SC therapy effects. On one hand, fresh SCs within 12 h after leaving the library will maintain good activity. On the other hand, the time window for IS treatment is concise. SC transplantation within 24 h after focal ischemia can notably reduce injury volume and improve motor dysfunction [168]. In contrast, SC transplantation after 24 h of IS will diminish the potential therapeutic effect [170]. Moreover, SC transplantation beyond this time frame does not exhibit a significant difference in infarct size compared to the MCAO group [168]. Although the intervention time window of SC therapy is longer than the intravenous injection of tissue plasminogen activators [168], it still faces the challenge of uncontrollable time frame. Whether for SCs themselves or stroke patients, these time frames are exceptionally challenging. Therefore, prolonging the time frame for maintaining SC activity and optimizing the time window of SCs treatment for IS is paramount.

## 5 Conclusion

The ability of SCs to treat IS has been widely reported and has shown excellent advantages compared to traditional methods, especially when implementing various mechanisms. Among them, modifying SCs to achieve better therapeutic effects is undoubtedly the present focus of researchers, and some breakthroughs have been made. However, despite some common issues in SC treatment, there are still many challenges regarding clinical transformation. Technologies, such as 3D microcarrier technology, have been rapidly developed to overcome SC clinical application limitations. We look forward to emerging technologies to accelerate the clinical transformation of SC therapy and enable SCs to be applied to patients. Furthermore, we expect to reveal SCs’ potential in treating IS, provide new ideas for SC transformation, and anticipate these challenges to be resolved as soon as possible.

## Conflicts of interest

The authors declare no competing financial interest.
